# Prediction of maternal complications and neonatal outcome in dichorionic diamniotic twins with fetal weight discordancy measured by ultrasonography

**DOI:** 10.1038/s41598-022-18864-8

**Published:** 2022-09-01

**Authors:** Hyun Mi Kim, Hyun-Hwa Cha, Won Joon Seong, Hye Jin Lee, Mi Ju Kim

**Affiliations:** Department of Obstetrics and Gynecology, Kyungpook National University Hospital, School of Medicine, Kyungpook National University, 41944 Daegu, Republic of Korea

**Keywords:** Medical research, Risk factors

## Abstract

This study aimed to determine the relationship between estimated fetal weight discordance by ultrasonography and maternal and neonatal outcomes in dichorionic diamniotic twin pregnancies. We conducted a retrospective review of the medical records of 106 twin pregnancies delivered at a single tertiary center between January 2011 and February 2020. At 20–24 and 28–32 weeks of gestation, participants were divided into two groups: discordant twins with an estimated fetal weight difference of more than 20% and concordant twins with a weight difference of less than 20%. Maternal complications and neonatal outcomes were compared between the two groups. Although the incidences of preeclampsia and placenta previa were significantly higher in discordant twins measured between 20 and 24 weeks, no statistical significance was found in neonatal outcomes. Delivery times were earlier, and neonatal weights were lower in discordant twins measured between 28 and 32 weeks. Neonatal outcomes such as ventilator use and neurodevelopment were also significantly different. Discordance in estimated fetal weight measured using ultrasonography between 20 and 24 weeks can be a risk factor for maternal preeclampsia and placenta previa, whereas discordance at 28–32 weeks may predict poor neonatal outcomes.

## Introduction

The incidence of multifetal pregnancies, including twins and triplets, is increasing worldwide. In the United States, approximately 33.3 births per 1000 pregnancies were twin pregnancies in 2009, a 76% increase since 1980^1^. Increasing maternal age and pregnancy through assisted reproductive techniques (ART) contribute to increased twin births^2,3^. The incidences of maternal pregnancy-related complications, neonatal morbidities, and mortality are higher in twin pregnancies than in singleton pregnancies. Preeclampsia occurs 2.6 times more frequently in twin pregnancies, which increases the risk of placental abruption and preterm delivery^4^. Twin pregnancies also increase maternal morbidity, such as gestational diabetes mellitus (GDM), postpartum hemorrhage (PPH), and operative delivery^5–8^. In neonates, twin pregnancies increase the risk of stillbirth by approximately five times^9^. In addition, neonatal morbidity and mortality increased, including a 6-fold increase in preterm births before 34 weeks^10^. Moreover, risks such as preterm birth (PTB) and stillbirth, congenital fetal anomaly, intrauterine growth restriction (IUGR), umbilical artery acidosis, neonatal intensive care unit (NICU) admission, and respiratory distress (RDS) are increased in discordant twins whose birth weights differ by more than 20%^2,11^. Neonatal morbidity increases by approximately 7.7 times in discordant twins and small-for-gestational-age (SGA) babies^2^. Fetal weight discordance after birth has been used to predict prognosis. The purpose of this study was to determine whether discordance of the estimated fetal weight (EFW) could predict neonatal outcomes of twin babies based on sonographic findings of the fetus performed during the prenatal examination. Additionally, the relationship between weight discordance and maternal pregnancy complications was investigated. This study used the EFW between 20–24 weeks and 28–32 weeks of gestation in twin pregnancies and compared maternal and neonatal outcomes between the discordant and concordant fetal weight groups.

## Results

This study included 106 twin pregnancies and 212 neonates. When weight discordance developed in twins between 20 and 24 weeks of pregnancy and between 28 and 32 weeks of gestation, the obstetric and neonatal outcomes were analyzed. Based on EFW between 20 and 24 weeks of gestation, pregnancies were divided into 95 concordant and 11 discordant weight twin pregnancies. According to EFW, between 28 and 32 weeks of gestation, pregnancies were divided into 90 concordant and 16 discordant weight twin pregnancies.

Table 1 shows a comparison of maternal characteristics and pregnancy-related complications in the concordant and discordant groups using weights at 20–24 weeks of gestation. Between the two groups, no statistically significant differences were observed in maternal age (32.58 ± 4.15 vs. 34.18 ± 5.03, *p* = 0.095) and body mass index (BMI) (pre-pregnancy, 22.33 ± 3.59 vs. 23.02 ± 3.35, *p* = 0.393). However, in the concordant group, significantly more nulliparous cases (77.89% vs. 63.64%, *p* < 0.001) and pregnancies through ART (70.53% vs. 63.64%, *p* = 0.034) were confirmed. No significant differences in GDM (17.89% vs. 0%, *p* = 0.063), threatened preterm labor (PTL) (46.32% vs. 36.36%, *p* = 0.508), premature rupture of the amniotic membrane (PROM) (14.74% vs. 9.09%, *p* = 0.695), and PPH (2.11% vs. 0.00%,* p* = 0.978) were observed between the two groups. However, the frequencies of preeclampsia (9.47% vs. 27.27%, *p* = 0.032) and placenta previa (2.11% vs. 18.81%, *p* = 0.002) were significantly higher in the discordant group than in the concordant group. No differences in the causes of delivery were observed between the two groups.

**Table 1:** Comparison of maternal characteristics and pregnancy-related complications between concordant and discordant twins at 20–24 weeks of gestation in DCDA twins.

	Concordant twin (n = 95)	Discordant twin (n = 11)	*p* value
Age (years)	32.58 ± 4.15	34.18 ± 5.03	0.095
Nulliparous, n (%)	74 (77.89%)	7 (63.64%)	< 0.001*
Prepregnant BMI (kg/m^2^)	22.33 ± 3.59	23.02 ± 3.35	0.393
BMI at delivery (kg/m^2^)	27.62 ± 3.88	28.25 ± 3.45	0.465
ART, n (%)	67 (70.53%)	7 (63.64%)	0.044*
Preeclampsia, n (%)	9 (9.47%)	3 (27.27%)	0.032*
GDM, n (%)	17 (17.89%)	0 (0.00%)	0.063
Placenta previa, n (%)	2 (2.11%)	2 (18.81%)	0.002*
Threatened PTL, n (%)	44 (46.32%)	4 (36.36%)	0.508
PROM, n (%)	4 (14.74%)	1 (9.09%)	0.692
PPH, n (%)	2 (2.11%)	0 (0.00%)	0.978
**Cause of delivery**			
Elective, n (%)	45 (47.37%)	5 (45.45%)	0.533
Spontaneous, n (%)	33 (34.74%)	3 (27.27%)	
Iatrogenic, n (%)	17 (17.89%)	3 (27.27%)	

DCDA, dichorionic diamniotic; BMI, body mass index; ART, artificial reproductive technique; GDM, gestational diabetes mellitus; PTL, preterm labor; PROM, premature rupture of the amniotic membrane; PPH, postpartum hemorrhage.

*p values of < 0.05 are shown in bold with an asterisk (*).

Table 2 shows the comparison of neonatal outcomes in both groups classified by weight at 20–24 weeks of gestation. No statistically significant differences were observed in gestational age (GA) at delivery (weeks, 35.70 ± 1.63 vs. 35.80 ± 1.37, *p* = 0.778), sex (male, 58.42% vs. 59.09%, *p* = 1.000), and birth weights (grams, 2322.11 ± 411.26 vs. 2323.64 ± 500.94, *p* = 0.987). Moreover, no differences were observed in the 1- and 5-min Apgar scores of 7 or less (13.68% vs. 4.55%, *p* = 0.193 and 0.00% vs. 4.55%, *p* = 0.160, respectively) and NICU hospitalization (64.21% vs. 68.18%, *p* = 0.894). The frequency of actual weight discordance at delivery (17.89% vs. 54.55%, *p* < 0.001) and developmental delay (3.68% vs. 18.18%, *p* = 0.017) were higher in the discordant group, whereas oxygen supply treatment (42.63% vs. 18.18%, *p* = 0.038) was higher in the concordant group. No significant differences were observed in neonatal morbidity, intubation, ventilator use, RDS, sepsis, patent ductus arteriosus (PDA), and retinopathy of prematurity (ROP). There was no statistically significant difference in neonatal outcomes between the discordant large and small, measured by ultrasonography between 20 and 24 weeks.

**Table 2:** Comparison of neonatal outcomes between concordant and discordant twins at 20–24 weeks of gestation in DCDA twins.

	Concordant twin (n = 190)	Discordant twin (n = 22)	*p* value
GA at delivery (weeks)	35.70 ± 1.63	35.80 ± 1.37	0.778
Sex, male, n (%)	111 (58.42%)	13 (59.09%)	1.000
Birthweight (grams)	2322.11 ± 411.26	2323.64 ± 500.94	0.987
Weight discordancy at delivery, n (%)	34 (17.89%)	12 (54.55%)	< 0.001*
Apgar score at 1 min (< 7), n (%)	26 (13.68%)	1 (4.55%)	0.193
Apgar score at 5 min (< 7), n (%)	0 (0.00%)	1 (4.55%)	0.160
NICU admission, n (%)	122 (64.21%)	15 (68.18%)	0.894
Neonatal morbidity, n (%)	47 (24.74%)	1 (4.55%)	0.061
Intubation, n (%)	9 (4.74%)	1 (4.55%)	0.944
Ventilator use (nasal cPAP), n (%)	59 (31.05%)	4 (18.18%)	0.315
Oxygen supply, n (%)	81 (42.63%)	4 (18.18%)	0.047*
Phototherapy, n (%)	39 (20.53%)	5 (22.73%)	1.000
Developmental delay, n (%)	7 (3.68%)	4 (18.18%)	0.017*

DCDA, dichorionic diamniotic; GA, gestational age; NICU, neonatal intensive care unit; cPAP, continuous positive airway pressure.

*p < 0.05, are shown in bold with an asterisk (*).

Table 3 shows the comparison of maternal characteristics and pregnancy-related complications in both groups, categorized using weight discordance at 28–32 weeks of gestation. In the discordant group, more ART trials (67.78% vs. 81.25%, *p* = 0.041) were performed, but no significant differences in maternal age, parity, or BMI were observed. Although preeclampsia was more prevalent in the discordant group (8.89% vs. 25.00%, *p* = 0.019), no significant difference in the frequency of placenta previa (3.33% vs. 6.25%, *p* = 0.768) was detected. Moreover, the frequency of iatrogenic cause of delivery was higher in the discordant group (14.44% vs. 43.75%, *p* < 0.001). No significant differences in the incidences of GDM, PROM, or threatened PTL were detected between the two groups.

**Table 3:** Comparison of maternal characteristics and pregnancy-related complications between concordant and discordant twins at 28–32 weeks of gestation in DCDA twins.

	Concordant twin (n = 90)	Discordant twin (n = 16)	*p *value
Age (years)	32.53 ± 4.05	33.94 ± 5.25	0.158
Nulliparous, n (%)	67 (74.44%)	14 (87.5%)	0.263
Prepregnant BMI (kg/m^2^)	22.40 ± 3.62	22.38 ± 3.31	0.976
BMI at delivery (kg/m^2^)	27.76 ± 3.89	27.23 ± 3.57	0.471
ART, n (%)	61 (67.78%)	13 (81.25%)	0.041*
Preeclampsia, n (%)	8 (8.89%)	4 (25.00%)	0.019*
GDM, n (%)	16 (17.78%)	1 (6.25%)	0.169
Placenta previa, n (%)	3 (3.33%)	1 (6.25%)	0.768
Threatened PTL, n (%)	40 (44.44%)	8 (50.00%)	0.697
PROM, n (%)	11 (12.22%)	4 (25.00%)	0.102
PPH, n (%)	2 (2.22%)	1 (3.12%)	1.000
**Cause of delivery**			
Elective, n (%)	48 (53.33%)	2 (12.50%)	< 0.001*
Spontaneous, n (%)	29 (32.22%)	7 (43.75%)	
Iatrogenic, n (%)	13 (14.44%)	7 (43.75%)	

DCDA, dichorionic diamniotic; BMI, body mass index; ART, artificial reproductive technique; GDM, gestational diabetes mellitus; PTL, preterm labor; PROM, premature rupture of the amniotic membrane; PPH, postpartum hemorrhage.

*p values of < 0.05 are shown in bold with an asterisk (*).

Table 4 shows the comparison of neonatal outcomes between the two groups categorized based on weight discordance at 28–32 weeks of gestation. Compared with the concordant group, the discordant group had earlier GA at delivery (35.93 ± 1.40 vs. 34.47 ± 2.07, *p* < 0.001), lower birth weights (2379.22 ± 370.29 vs. 2001.88 ± 533.51, *p* < 0.001), and higher NICU admission (60.56% vs. 87.50%, *p* = 0.006). Moreover, discordant twins required intubation (2.78% vs. 15.62%,* p* = 0.007), ventilator use (25.56% vs. 53.12%, *p* = 0.003), oxygen supply therapy (36.11% vs. 62.50%, *p* = 0.009), and phototherapy due to hyperbilirubinemia (16.67% vs. 43.75%, *p* = 0.001) more often than concordant twins. Moreover, developmental delays between 1 and 2 years after birth were more frequent in the discordant group (2.22% vs. 21.88%, *p* < 0.001). In the discordant group, there was no statistically significant difference in neonatal outcomes between the discordant large and discordant small neonates. However, in discordant small babies, lower 1 min Apgar scores and higher NICU admission rates were observed.

**Table 4:** Comparison of neonatal outcomes between the concordant and discordant twins at 28–32 weeks of gestation in DCDA twins.

	Concordant twin (n = 180)	Discordant twin (n = 32)	*p* value
GA at delivery (weeks)	35.93 ± 1.40	34.47 ± 2.07	< 0.001*
Sex, male, n (%)	104 (57.78%)	20 (62.50%)	0.760
Birthweight (gram)	2379.22 ± 370.29	2001.88 ± 533.51	< 0.001*
Weight discordancy at delivery, n (%)	22 (12.22%)	24 (75.00%)	< 0.001*
Apgar score at 1 min (< 7), n (%)	19 (10.56%)	8 (25.00%)	0.049*
Apgar score at 5 min (< 7), n (%)	0 (0.00%)	1 (3.12%)	0.328
NICU admission, n (%)	109 (60.56%)	28 (87.50%)	0.006*
Neonatal morbidity, n (%)	36 (20.00%)	12 (37.50%)	0.051
Intubation, n (%)	5 (2.78%)	5 (15.62%)	0.007*
Ventilator use (nasal cPAP), n (%)	46 (25.56%)	17 (53.12%)	0.003*
Oxygen supply, n (%)	65 (36.11%)	20 (62.50%)	0.009*
Phototherapy, n (%)	30 (16.67%)	14 (43.75%)	0.001*
Developmental delay, n (%)	4 (2.22%)	7 (21.88%)	< 0.001*

DCDA, dichorionic diamniotic; GA, gestational age; NICU, neonatal intensive care unit; cPAP, continuous positive airway pressure.

*p values of < 0.05 are shown in bold with an asterisk (*).

**Figure 1:**
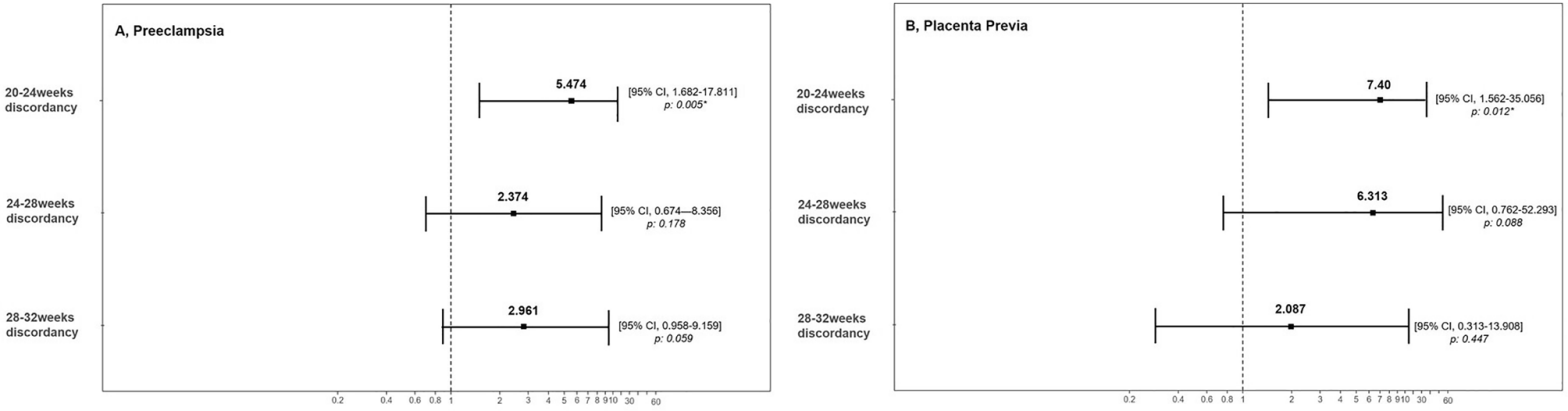
Regression analysis of the relationship between twin discordancy and risk of maternal obstetric complications after adjustment for the confounding factors of maternal age, pre-pregnancy BMI, BMI at delivery, and gestational age at delivery. (**A**) Preeclampsia, (**B**) Placenta previa. Odds ratios (ORs) and 95% confidence intervals (CIs) were calculated, and a *p* value of < 0.05 was considered to be statistically significant. BMI, body mass index.

Figure 1 shows the results of the regression analysis to determine whether EFW discordance in twin pregnancy could predict the occurrence of preeclampsia and placenta previa. The results were calculated after adjusting for the confounding factors of maternal age, pre-pregnancy BMI, BMI at delivery, and GA at delivery. The incidence of preeclampsia was confirmed by an odds ratio (OR) of 5.474 (95% confidence interval (CI), 1.682–17.811; *p* = 0.005) for discordance between 20 and 24 weeks of gestation and an OR of 2.961 (95% CI 0.958–9.159; *p* = 0.059) for discordance between 28 and 32 weeks of gestation. Moreover, placenta previa was predicted by an OR of 7.400 (95% CI 1.562–35.056; *p* = 0.012) for discordance between 20 and 24 weeks of gestation, and an OR of 2.087 (95% CI 0.313–13.908; *p* = 0.447) for discordant twins between 28 and 32 weeks.

**Figure 2:**
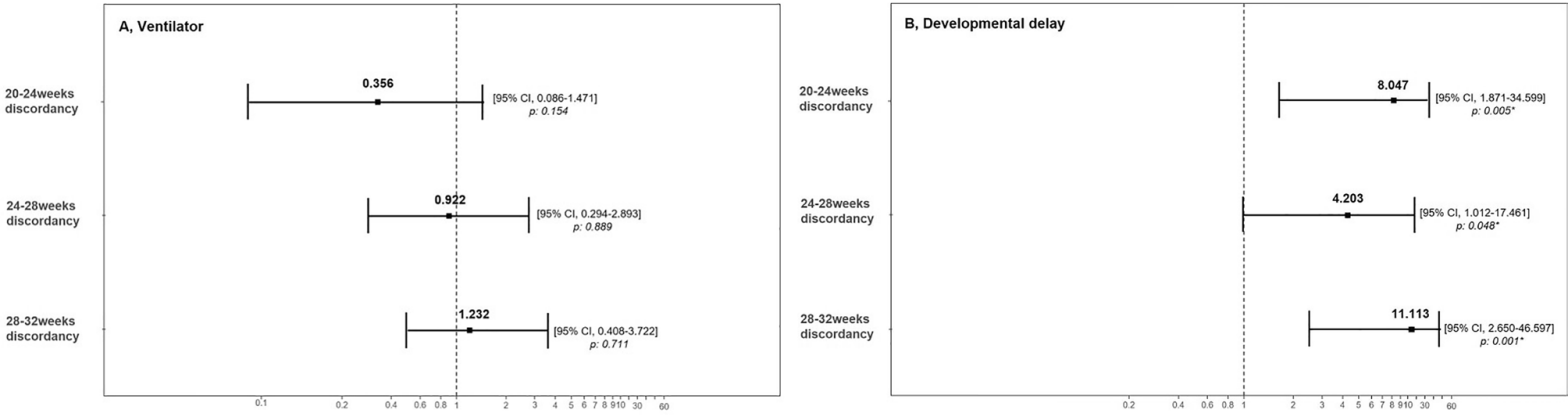
Regression analysis of the relationship between twin discordancy and risk of neonatal outcomes after adjustment for the confounding factors of neonatal birth weight, gestational age at delivery, and NICU admission. (**A**) Intubation, (**B**) Developmental delay. Odds ratios (ORs) and 95% confidence intervals (CIs) were calculated and a *p* value of < 0.05 was considered to be statistically significant. NICU, neonatal intensive care unit.

Figure 2 shows the results of the regression analysis to determine neonatal outcomes, including the use of a ventilator and the presence of developmental delay based on discordance during pregnancy. The results were calculated after adjusting for confounding factors of neonatal birth weight, GA at delivery, and NICU admission. The use of a ventilator was predicted by the OR of discordance between 20 and 24 weeks as 0.356 (95% CI 0.086–1.471; *p* = 0.154) and the OR of discordance between 28 and 32 weeks as 1.232 (95% CI 0.408–3.722; *p* = 0.711). Developmental delay was predicted by an OR of 8.047 (95% CI 0.874–34.599; *p* = 0.005) for discordance between 20 and 24 weeks and an OR of 11.113 (95% CI 2.650–46.597; *p* = 0.001) for discordance between 28 and 32 weeks.

## Discussion

Twin discordance, a twin-specific condition, has a total incidence of 10–15%, and the prevalence does not differ according to chorionicity^12,13^. The reasons for the discordance in twin pregnancies differ depending on chorionicity. In dichorionic twins, discordance is due to potential differences in genetic growth and placental dysfunction, which account for the demands for oxygen and nutrition, which increase as the fetus grows. Incomplete conversion of uteroplacental circulation induces ischemic changes in placental tissue, which induces oxidative stress, apoptosis, and, subsequently, fetal growth differences^14–20^. In monochorionic twins, discordance arises because of the differences in the sharing of one placenta^20^. The earlier the onset of discordance, the lower the long-term survival of the fetus due to the occurrence of twin-to-twin transfusion syndrome (TTTS)^21,22^. Twin discordance is related to poor neonatal prognoses such as respiratory complications, stillbirths, SGA, and maternal morbidity. However, to our knowledge, few studies have investigated the weight discordance between two fetuses using ultrasonography measured during pregnancy and determined the relationship between weight discordance and maternal pregnancy-related complications and neonatal morbidities. The maternal and neonatal outcomes of concordant and discordant twins were compared at both gestational periods.

Discordant twins between 20 and 24 weeks of gestation were found to have more complications related to maternal abnormal placentation, such as preeclampsia and placenta previa, compared with concordant twins. Preeclampsia is more common in twin pregnancies than in singleton pregnancies^23^. Among dichorionic twins, the incidence of preeclampsia was higher in discordant birth weight twins than in concordant twins. In addition, studies have shown that the more severe the weight discordance, the greater the occurrence of preeclampsia and gestational hypertension^24^. These findings are consistent with the results of this study: discordance during pregnancy predicted the occurrence of maternal preeclampsia. The pathophysiology of the relationship between discordance and preeclampsia during pregnancy is unknown. However, there are several possible explanations. Discordance during pregnancy is either a cause or effect of placental ischemia or hypoxia^25^. Placental hypoperfusion caused by hypoxia may activate maternal endothelial dysfunction, which manifests as symptoms of vascular constriction and high blood pressure^24,26^. In addition, placental hypoperfusion affects maternal angiogenic factors such as soluble fms-like tyrosine kinase-1 (sFlt-1) and placental growth factor, which are related to the development of preeclampsia^27^. Placental abnormalities such as location and weight of the placenta may also affect twin discordance and fetal growth^28^. A previous meta-analysis of singletons showed an association between fetal growth and placenta previa^29^. Most cases of placenta previa diagnosed on ultrasonography in the routine mid-trimester resolve before late gestation. However, placenta previa persisting until delivery may be related to placental hypoperfusion. Abnormal placentation such as placenta previa may induce placental hypoperfusion, which may cause discordance.

When comparing concordant and discordant twins using weight discordance between 28 and 32 weeks of gestation, discordant twins had a higher tendency to develop preeclampsia, but no significant differences were observed in the incidence of placenta previa. There were no significant differences in the incidence of GDM, PTL, or cause of delivery. No significant differences in gestational age at delivery and birth weight were observed when discordance was measured at 20 and 24 weeks of gestation. Except for developmental delay 1–2 years after birth, neonatal morbidities and complications also showed no significant differences. However, when comparing concordant and discordant twins using weight discordancy between 28 and 32 weeks of gestation, worse neonatal outcomes were observed in discordant twins. In the discordant group, the average GA at delivery was approximately 1 week earlier, and the fetuses tended to have lower birth weights. Delivery was more likely to be an induction delivery or cesarean section for medical reasons rather than an elective or scheduled delivery for discordant twins. Neonatal outcomes were worse, resulting in more NICU hospitalizations, and the presence of complications, such as morbidities, need for intubation, use of a ventilator, and phototherapy, tended to be higher in discordant twins. Developmental delays between 1 and 2 years of age also tended to be higher in the discordant group. Halling et al. reported that smaller discordant twins have more developmental disorders in cognition, language, and motor skills than larger twins do. However, PTB before 33 weeks of gestation had a greater effect on developmental delay than did discordance^28^. This study is different from other studies in that the study was limited to cases that were delivered after 32 weeks of gestation, and the predicted weight during pregnancy, not the actual birth weight, was used. Weight discordance after the third trimester of pregnancy is not due to the abnormal placentation of the mother, but rather the cause and effect of stress or insult to the fetus in the uterus due to various factors. Thus, the long-term intrauterine environment affects not only short-term outcomes such as oxygen therapy, which may appear immediately after delivery, but also long-term outcomes, such as developmental delay, that may appear 1–2 years after the birth of the fetus. In addition, weight discordance that occurs between 28 and 32 weeks of gestation is considered a sign that delivery is required and may lead to preterm births, which may affect short and long-term perinatal outcomes of the fetus.

According to the regression analyses of fetal weights at 20–24 weeks, preeclampsia is 5.5 times more likely to occur, and placenta previa is 7.4 times more likely to occur in discordant weight twins than in concordant weight twins. However, the discordance between 28 and 32 weeks was less significant as a diagnostic predictor. Therefore, sonographic findings in the second trimester of pregnancy may predict an abnormal placenta. In contrast, discordant weight fetuses measured between 28 and 32 weeks are 1.2 times more likely to use a ventilator and 11 times more likely to have developmental delays after birth after adjusting for confounding factors. Thus, discordance at 28–32 weeks was a predictor of short and long-term neonatal outcomes. In the discordant twin between 20 and 24 weeks, GA at delivery, birth weight, and short-term outcomes were similar to those of the concordant twin. However, there was a significant difference in the developmental delay. Intrauterine insult, which can induce discordance in early pregnancy, affects fetal neurodevelopment, but more research is needed.

This study has several limitations. First, ultrasonography was subjective. However, to reduce intra-observer bias, sonographic values were measured three or more times in a row. Moreover, to reduce interobserver bias, one obstetrician performed sonographic measurements continuously according to the number of weeks of pregnancy. An expert reviewed the sonographic images and re-evaluated whether the images were appropriate. Second, the sample size was small. The study included only healthy dichorionic diamniotic (DCDA) twin mothers who had complete medical records and did not have fetal death, fetal anomalies, or twin-specific complications. Further research with a larger sample size is warranted. Finally, selective IUGR was not considered in this study. In this study, only the weight difference between the two fetuses of DCDA twins was analyzed, and the effect is unknown; therefore, it should be further addressed in future studies.

In twin pregnancies, discordance using the estimated weight of the fetus between 20 and 24 weeks of gestation can predict the possibility of abnormal placentation, such as preeclampsia or placenta previa, whereas discordance between 28 and 32 weeks of gestation can predict a higher risk of short and long-term poor neonatal outcomes.

## Methods

**Figure 3:**
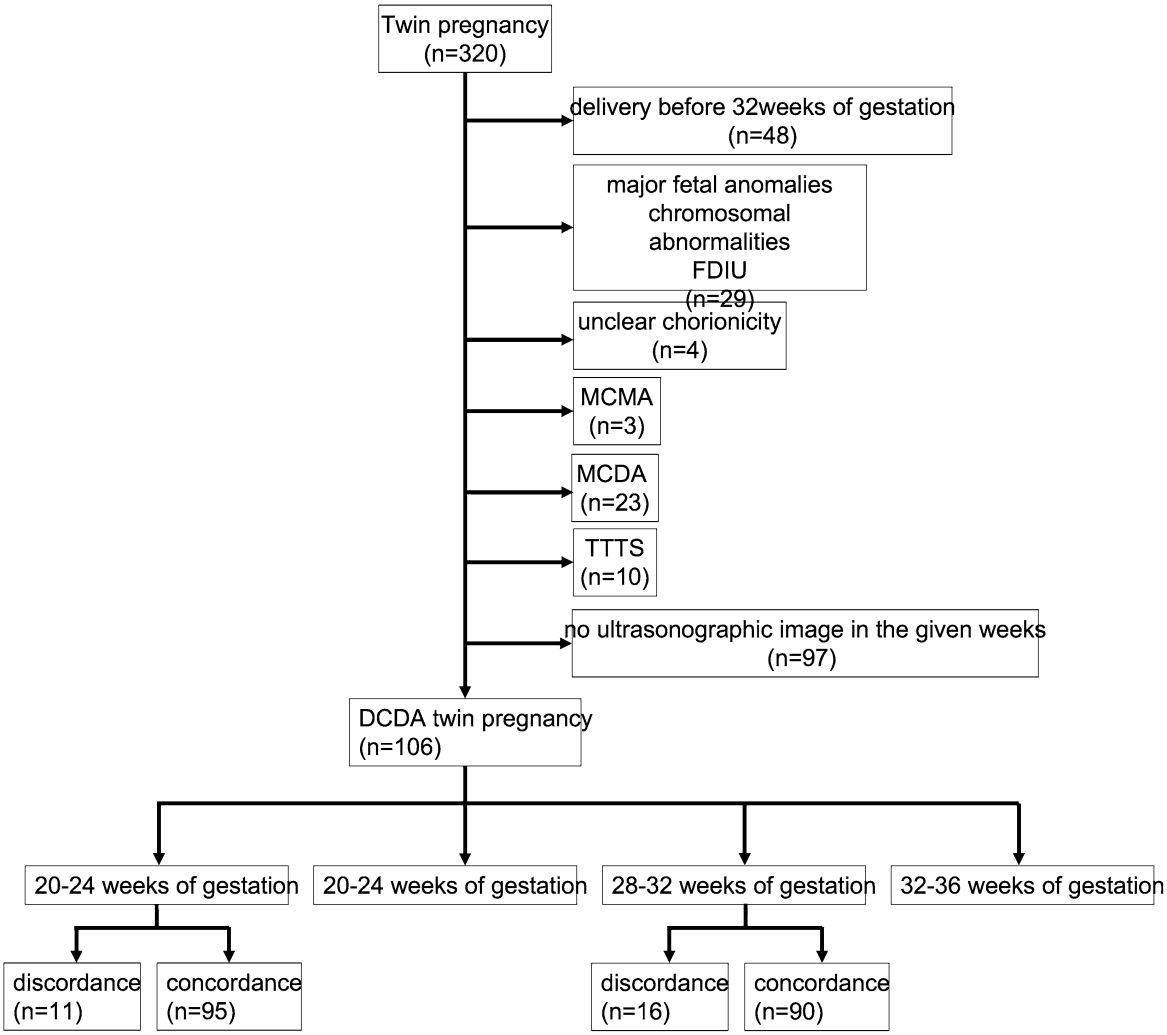
Flowchart for the review of studies. FDIU, fetal death in uterus; MCMA, monochorionic monoamniotic; MCDA, monochorionic diamniotic; TTTS, twin-to-twin transfusion syndrome; DCDA, dichorionic diamniotic.

The medical records of 320 patients with twin pregnancies who delivered at Kyungpook National University Chilgok Hospital in Daegu, South Korea between January 2011 and February 2020 were retrospectively reviewed. Based on 32 weeks of gestation, when lung maturation was expected in twins, 48 ​​pregnancies delivered before 32 weeks of gestation were excluded^30^. This was done to minimize the impact of neonatal outcomes due to prematurity. An additional 29 women were excluded because of major fetal anomalies, chromosomal abnormalities, or fetal death in utero. Four women with unclear chorionicity, 3 with monochorionic monoamniotic (MCMA) twins, 23 with monochorionic diamniotic (MCDA) twins, and 10 with TTTS were also excluded. When chorionicity in the medical records and placental pathologic findings after delivery were different, the pathologic result was followed. Thus, 117 pregnancies were excluded, and 203 twin pregnancies were reviewed. Fetal ultrasonography (Samsung Medison WS 80A, Korea) reports between 20 and 24 weeks of gestation and 28 and 32 weeks of gestation were reviewed. The weight was measured three times per fetus, and the average value was taken as the EFW. Ninety-seven pregnancies were delivered; however, sonographic measurements were not performed between 20 and 24 weeks of gestation or 28 and 32 weeks of gestation and were excluded from the study. Thus, the final number of pregnancies included in the analysis was 106. A flowchart illustrating the study plan is shown in Fig. 3.

Weight discordance was measured using the EFW of the two fetuses using the Hadlock formula, which was multiplied by 100 after dividing the difference between the weights of the two fetuses by the expected weight of the large fetus, as defined in previous studies^3^. If the value was > 20%, the fetuses were classified as discordant twins, and if the value was less than 20%, the fetuses were classified as concordant twins according to the consensus of the American College of Obstetricians and Gynecologists (ACOG) and the International Society of Obstetrics and Gynecology (ISUOG)^31^. In DCDA twins, discordance was distinguished only by weight difference, and selective fetal growth restriction (sFEG), which is IUGR in one of the two fetuses, was not considered^32^. Maternal characteristics such as age at delivery, parity, pre-pregnancy BMI, use of ARTs, and pregnancy-related complications such as preeclampsia, placenta previa, GDM, threatened PTL, and PPH were compared. According to the International Society for the Study of Hypertension in Pregnancy, preeclampsia was defined as a blood pressure of 140/90 mmHg or higher and proteinuria of 300 mg or higher for 24 h after 20 weeks of gestation^33^. A 50-gm glucose tolerance test was performed as a screening test for GDM between 24 and 28 weeks of gestation, and a 100-gm glucose tolerance test was performed when the value was > 140 mg/dL. GDM was diagnosed according to the Carpentre–Coustan criteria^34^. Placenta previa was defined by evaluating the location of the placenta through ultrasonography immediately before delivery, including placenta previa totalis, placenta previa partialis, placenta previa marginalis, and a low-lying placenta. Threatened PTL was defined as inpatient conservative treatment before 37 weeks of gestation owing to regular uterine contractions and a short cervical length of less than 25 mm. PPH was defined as bleeding of more than 1 L or getting a transfusion after delivery because of anemia. Deliveries scheduled on a non-event date were classified as elective, deliveries due to labor pain as spontaneous, and emergency deliveries due to preeclampsia or poor fetal condition as iatrogenic. A comparison was made between the 20th and 36th weeks of pregnancy, divided into 4-week intervals. The 32–36 weeks of gestation, where there was no significant difference between EFW and birth weight, were excluded. There was no difference in EFW between 24–28 and 28–32 weeks of gestation. The early gestational weeks could be affected by genetics, and hence, 24–28 weeks of gestation were excluded from the analysis. Maternal complications and neonatal outcomes were analyzed at 20–24 and 28–32 weeks of gestation. To compare the neonatal outcomes of discordant and concordant pregnancies, GA at delivery, neonatal birth weight, 1-min and 5-min Apgar scores, NICU admission, neonatal morbidity, and mortality were analyzed. The morbidities included RDS, intraventricular hemorrhage (IVH), periventricular leukomalacia (PVL), necrotizing enterocolitis (NEC), and sepsis. We reviewed the rate of developmental delay in cases that had fine and gross motor disturbances observed through physical examination and the Bayley Scale, and in those that required rehabilitation 1–2 years after birth. This study was approved by the Institutional Review Board (IRB) of Chilgok Kyungpook National University Hospital (IRB No. KNUCH 2020–06-015). All methods were performed in accordance with the relevant guidelines and regulations. The requirement for informed consent was waived by Institutional Review Board (IRB) of Chilgok Kyungpook National University Hospital because of the retrospective nature of the review of medical records. This study was approved by the Institutional Review Board (IRB) of the Chilgok Kyungpook National University Hospital.

### Statistical analysis

All data were analyzed with IBM SPSS Statistics for Windows, version 26.0, (IBM Corp., Armonk, N.Y., USA) and R version 4.0.0 (Vienna, Austria; www.r-project.org/). The results of the concordant and discordant groups were compared using the Mann–Whitney U test for continuous numerical data, whereas the χ^2^ test was used for binary categorical data. Data is presented as mean ± standard deviation for continuous variables with normal distributions and numbers (percentages) for binary categorical data. A *p* value of < 0.05 was considered to be statistically significant. Logistic regression analyses were conducted to identify independent variables predictive of maternal complications and neonatal outcomes among concordant and discordant twins. ORs and 95% confidence intervals (CIs) were calculated.

### Ethics approval and consent to participate

This study was approved by the Institutional Review Board (IRB) of Chilgok Kyungpook National University Hospital (IRB No. KNUCH 2020-06-015). Informed consent was not obtained from the study participants due to the retrospective nature of the review of medical records.

## Data Availability

The datasets used and analyzed during the current study are available from the corresponding author upon reasonable request.
